# 1-Mesitylmethyl-1*H*benzotriazole 3-oxide

**DOI:** 10.1107/S1600536810004824

**Published:** 2010-02-13

**Authors:** B. Ravindran Durai Nayagam, Samuel Robinson Jebas, J. Shakina, R. Murugesan., Dieter Schollmeyer

**Affiliations:** a Department of Chemistry, Popes College, Sawyerpuram 628251, Tamilnadu, India; b Department of Physics, Sethupathy Government Arts College, Ramanathapuram 623502, Tamilnadu, India; c Department of Chemistry, Sarah Tucker College, Tirunelveli 627007, Tamilnadu, India; d Department of Chemistry, T.D.M.N.S. College, T. Kallikulam, Tamilnadu, India; e Institut für Organische Chemie, Universität Mainz, Duesbergweg 10-14, 55099 Mainz, Germany

## Abstract

In the title compound, C_16_H_17_N_3_O, the benzotriazole ring forms a dihedral angle of 77.25 (6)° with the phenyl ring. The benzotriazole ring is essentially planar with a maximum deviation of 0.012 (19) Å. Weak inter­molecular C—H⋯O hydrogen bonds form *R*_2_^2^(10) motifs. The crystal packing is consolidated by π—π inter­actions with centroid–centroid distances of 3.5994 (12) Å together with very weak C—H⋯π inter­actions.

## Related literature

For bond-length data, see: Allen *et al.* (1987 ▶). For graph-set analysis of hydrogen bonding, see: Bernstein *et al.* (1995 ▶).
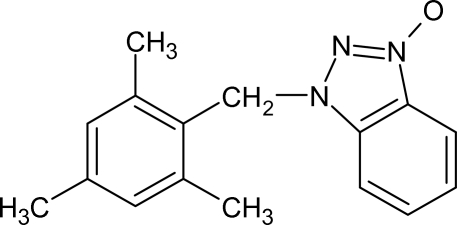


## Experimental

### 

#### Crystal data

C_16_H_17_N_3_O
*M* *_r_* = 267.33
Monoclinic, 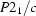

*a* = 8.6269 (19) Å
*b* = 7.3422 (4) Å
*c* = 21.890 (5) Å
β = 103.133 (11)°
*V* = 1350.2 (4) Å^3^
*Z* = 4
Cu *K*α radiation
μ = 0.67 mm^−1^
*T* = 193 K
0.35 × 0.20 × 0.10 mm

#### Data collection

Enraf–Nonius CAD-4 diffractometer
Absorption correction: ψ scan (*CORINC*; Draeger & Gattow (1971 ▶) *T*_min_ = 0.799, *T*_max_ = 0.936
2722 measured reflections
2545 independent reflections
2243 reflections with *I* > 2σ(*I*)
*R*_int_ = 0.091
3 standard reflections every 60 min  intensity decay: 2%

#### Refinement

*R*[*F* ^2^ > 2σ(*F* ^2^)] = 0.046
*wR*(*F* ^2^) = 0.132
*S* = 1.09
2546 reflections
184 parameters
H-atom parameters constrained
Δρ_max_ = 0.23 e Å^−3^
Δρ_min_ = −0.26 e Å^−3^

### 

Data collection: *CAD-4 EXPRESS* (Enraf–Nonius, 1994 ▶); cell refinement: *CAD-4 EXPRESS*; data reduction: *CORINC* (Draeger & Gattow, 1971 ▶); program(s) used to solve structure: *SHELXS97* (Sheldrick, 2008 ▶); program(s) used to refine structure: *SHELXL97* (Sheldrick, 2008 ▶); molecular graphics: *SHELXTL* (Sheldrick, 2008 ▶); software used to prepare material for publication: *SHELXTL* and *PLATON* (Spek, 2009 ▶).

## Supplementary Material

Crystal structure: contains datablocks global, I. DOI: 10.1107/S1600536810004824/bt5182sup1.cif

Structure factors: contains datablocks I. DOI: 10.1107/S1600536810004824/bt5182Isup2.hkl

Additional supplementary materials:  crystallographic information; 3D view; checkCIF report

## Figures and Tables

**Table 1:** Hydrogen-bond geometry (Å, °) *Cg*3 is the centroid of the C12–C17 ring.

*D*—H⋯*A*	*D*—H	H⋯*A*	*D*⋯*A*	*D*—H⋯*A*
C6—H6⋯O10^i^	0.95	2.35	3.190 (2)	147
C16—H16⋯O10^ii^	0.95	2.58	3.506 (2)	165
C18—H18*A*⋯*Cg*3^iii^	0.98	2.98	3.810 (18)	144

Symmetry codes: (i) 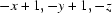; (ii) 
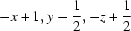
; (iii) 
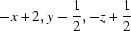
.

**Fig. 1.**
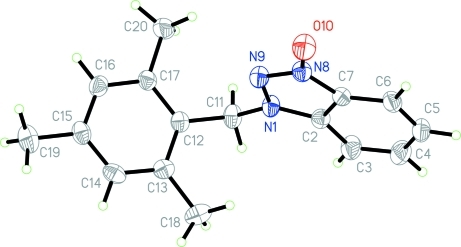
The molecular structure of the title compound, showing 50% probability displacement ellipsoids and the atom numbering scheme.

## supplementary crystallographic information

### Comment

The asymmetric unit of (I) comprises of one molecule of the title compound (Fig 1). The bond lengths and angles are found to have normal values (Allen *et al.*, 1987). The benzotriazole ring is essentially planar with the maximum deviation from planarity being 0.012 (19) Å for atom C5. The mean plane of the benzotriazole ring (N1/N9/N8/C2—C7) forming a dihedral angle of 77.25 (6) Å with the mean plane of the phenyl ring (C12—C17). An intermolecular weak C—H···O hydrogen bonding generates a ring of motif *R*_2_^2^(10) (Bernstein *et al.*, 1995)

The crystal packing  is stabilized by π—π stacking interactions [*Cg*1—*Cg*2^i^= of 3.5994 (12) Å; *Cg*1: (N1/N9/N8/C7/C2); *Cg*2:(C2—C): Symmetry code:(i) 1-*X*, –Y, –*Z*] together with weak C—H···π interactions.

### Experimental

A mixture of mono(bromomethyl)mesitylene (0.213 g, 1 mmol) and sodium salt of 1-Hydroxybenzotriazole (0.157,1 mmol) in ethanol (20 ml) was heated at 333 K with stirring for 30 min. The compound formed was filtered off, and dried. The compound was dissolved in ethanol and chloroform (1: 1v/v) and allowed to undergo slow evaporation. Colourless block shaped crystals  were obtained after a week.

### Refinement

All the H atoms were positioned geometrically (C—H=0.95 Å (aromatic); C—H=0.98 (methyl) or C—H=0.99 Å (methylene) and refined using a riding model with, *U*_iso_(H)=1.2U_equ_(C, methylene) and 1.5U_equ_(C_methyl_). A rotating group model was used for the methyl groups.

### Crystal data

**Table:**

C_16_H_17_N_3_O	*F*(000) = 568
*M**_r_* = 267.33	*D*_x_ = 1.315 Mg m^−^^3^
Monoclinic, *P*2_1_/*c*	Cu *K*α radiation, λ = 1.54178 Å
Hall symbol: -P 2ybc	Cell parameters from 25 reflections
*a* = 8.6269 (19) Å	θ = 35–48°
*b* = 7.3422 (4) Å	µ = 0.67 mm^−^^1^
*c* = 21.890 (5) Å	*T* = 193 K
β = 103.133 (11)°	Block, colourless
*V* = 1350.2 (4) Å^3^	0.35 × 0.20 × 0.10 mm
*Z* = 4	

### Data collection

**Table:**

Enraf–Nonius CAD-4 diffractometer	2243 reflections with *I* > 2σ(*I*)
Radiation source: rotating anode	*R*_int_ = 0.091
graphite	θ_max_ = 69.9°, θ_min_ = 4.2°
ω/2θ scans	*h* = 0→10
Absorption correction: ψ scan (CORINC; Draeger & Gattow (1971)	*k* = −8→0
*T*_min_ = 0.799, *T*_max_ = 0.936	*l* = −26→25
2722 measured reflections	3 standard reflections every 60 min
2545 independent reflections	intensity decay: 2%

### Refinement

**Table:**

Refinement on *F*^2^	Primary atom site location: structure-invariant direct methods
Least-squares matrix: full	Secondary atom site location: difference Fourier map
*R*[*F*^2^ > 2σ(*F*^2^)] = 0.046	Hydrogen site location: inferred from neighbouring sites
*wR*(*F*^2^) = 0.132	H-atom parameters constrained
*S* = 1.09	*w* = 1/[σ^2^(*F*_o_^2^) + (0.0705*P*)^2^ + 0.4335*P*] where *P* = (*F*_o_^2^ + 2*F*_c_^2^)/3
2546 reflections	(Δ/σ)_max_ < 0.001
184 parameters	Δρ_max_ = 0.23 e Å^−^^3^
0 restraints	Δρ_min_ = −0.26 e Å^−^^3^

### Special details

**Table:**

Geometry. All esds (except the esd in the dihedral angle between two l.s. planes) are estimated using the full covariance matrix. The cell esds are taken into account individually in the estimation of esds in distances, angles and torsion angles; correlations between esds in cell parameters are only used when they are defined by crystal symmetry. An approximate (isotropic) treatment of cell esds is used for estimating esds involving l.s. planes.
Refinement. Refinement of *F*^2^ against ALL reflections. The weighted *R*-factor wR and goodness of fit *S* are based on *F*^2^, conventional *R*-factors *R* are based on *F*, with *F* set to zero for negative *F*^2^. The threshold expression of *F*^2^ > σ(*F*^2^) is used only for calculating *R*-factors(gt) etc. and is not relevant to the choice of reflections for refinement. *R*-factors based on *F*^2^ are statistically about twice as large as those based on *F*, and *R*- factors based on ALL data will be even larger.

### Fractional atomic coordinates and isotropic or equivalent isotropic displacement parameters (Å^2^)

**Table:**

	*x*	*y*	*z*	*U*_iso_*/*U*_eq_	
N1	0.70466 (16)	−0.01341 (18)	0.11411 (6)	0.0277 (3)	
C2	0.71052 (18)	0.0150 (2)	0.05338 (7)	0.0272 (3)	
C3	0.7611 (2)	−0.0918 (2)	0.00798 (8)	0.0369 (4)	
H3	0.7999	−0.2123	0.0167	0.044*	
C4	0.7513 (2)	−0.0124 (3)	−0.04902 (8)	0.0411 (4)	
H4	0.7846	−0.0801	−0.0808	0.049*	
C5	0.6933 (2)	0.1673 (3)	−0.06290 (8)	0.0364 (4)	
H5	0.6900	0.2166	−0.1033	0.044*	
C6	0.64222 (18)	0.2710 (2)	−0.01957 (7)	0.0317 (4)	
H6	0.6023	0.3909	−0.0286	0.038*	
C7	0.65231 (17)	0.1899 (2)	0.03868 (7)	0.0262 (3)	
N8	0.61389 (15)	0.25395 (18)	0.09300 (6)	0.0285 (3)	
N9	0.64505 (16)	0.13267 (18)	0.13845 (6)	0.0294 (3)	
O10	0.56088 (16)	0.41370 (17)	0.10077 (6)	0.0416 (3)	
C11	0.7402 (2)	−0.1818 (2)	0.15108 (7)	0.0308 (4)	
H11A	0.8203	−0.2529	0.1352	0.037*	
H11B	0.6421	−0.2562	0.1450	0.037*	
C12	0.80200 (18)	−0.1471 (2)	0.22014 (7)	0.0258 (3)	
C13	0.96499 (17)	−0.1154 (2)	0.24416 (7)	0.0278 (3)	
C14	1.01987 (18)	−0.0911 (2)	0.30840 (8)	0.0307 (4)	
H14	1.1305	−0.0728	0.3249	0.037*	
C15	0.9183 (2)	−0.0928 (2)	0.34912 (7)	0.0311 (4)	
C16	0.75646 (19)	−0.1203 (2)	0.32423 (7)	0.0307 (4)	
H16	0.6850	−0.1197	0.3515	0.037*	
C17	0.69733 (18)	−0.1485 (2)	0.26051 (7)	0.0272 (3)	
C18	1.0814 (2)	−0.1051 (2)	0.20228 (9)	0.0396 (4)	
H18A	1.0922	−0.2256	0.1844	0.059*	
H18B	1.1852	−0.0648	0.2269	0.059*	
H18C	1.0424	−0.0181	0.1683	0.059*	
C19	0.9812 (2)	−0.0637 (3)	0.41845 (8)	0.0460 (5)	
H19A	0.9922	−0.1816	0.4400	0.069*	
H19B	0.9070	0.0132	0.4348	0.069*	
H19C	1.0853	−0.0039	0.4257	0.069*	
C20	0.52065 (19)	−0.1764 (3)	0.23637 (8)	0.0393 (4)	
H20A	0.4771	−0.0771	0.2076	0.059*	
H20B	0.4684	−0.1772	0.2717	0.059*	
H20C	0.5017	−0.2929	0.2141	0.059*	

### Atomic displacement parameters (Å^2^)

**Table:**

	*U*^11^	*U*^22^	*U*^33^	*U*^12^	*U*^13^	*U*^23^
N1	0.0360 (7)	0.0243 (7)	0.0232 (6)	0.0026 (5)	0.0075 (5)	0.0011 (5)
C2	0.0279 (7)	0.0279 (8)	0.0258 (7)	−0.0008 (6)	0.0060 (6)	−0.0003 (6)
C3	0.0467 (9)	0.0346 (9)	0.0297 (8)	0.0077 (7)	0.0096 (7)	−0.0025 (7)
C4	0.0477 (10)	0.0471 (11)	0.0304 (9)	0.0048 (8)	0.0131 (7)	−0.0050 (8)
C5	0.0394 (9)	0.0446 (10)	0.0249 (8)	−0.0020 (8)	0.0064 (7)	0.0047 (7)
C6	0.0304 (8)	0.0338 (9)	0.0290 (8)	0.0001 (6)	0.0028 (6)	0.0053 (7)
C7	0.0242 (7)	0.0283 (8)	0.0255 (7)	−0.0007 (6)	0.0042 (6)	−0.0001 (6)
N8	0.0315 (7)	0.0251 (7)	0.0284 (6)	0.0036 (5)	0.0060 (5)	0.0007 (5)
N9	0.0364 (7)	0.0255 (7)	0.0270 (6)	0.0032 (5)	0.0083 (5)	−0.0002 (5)
O10	0.0574 (8)	0.0284 (6)	0.0406 (7)	0.0164 (5)	0.0141 (6)	0.0016 (5)
C11	0.0442 (9)	0.0216 (8)	0.0261 (8)	0.0000 (6)	0.0070 (7)	0.0023 (6)
C12	0.0312 (8)	0.0195 (7)	0.0263 (7)	0.0013 (6)	0.0057 (6)	0.0023 (6)
C13	0.0304 (8)	0.0200 (7)	0.0344 (8)	0.0026 (6)	0.0099 (6)	0.0005 (6)
C14	0.0262 (7)	0.0231 (8)	0.0397 (9)	0.0012 (6)	0.0011 (6)	−0.0001 (6)
C15	0.0384 (8)	0.0250 (8)	0.0274 (8)	0.0030 (6)	0.0023 (6)	0.0019 (6)
C16	0.0349 (8)	0.0296 (8)	0.0295 (8)	0.0035 (6)	0.0112 (6)	0.0061 (6)
C17	0.0285 (7)	0.0250 (8)	0.0275 (8)	0.0003 (6)	0.0053 (6)	0.0065 (6)
C18	0.0378 (9)	0.0342 (9)	0.0523 (11)	−0.0014 (7)	0.0217 (8)	−0.0040 (8)
C19	0.0543 (11)	0.0473 (11)	0.0313 (9)	0.0008 (9)	−0.0006 (8)	−0.0014 (8)
C20	0.0290 (8)	0.0495 (11)	0.0383 (9)	−0.0042 (7)	0.0053 (7)	0.0096 (8)

### Geometric parameters (Å, °)

**Table:**

N1—N9	1.3502 (18)	C12—C13	1.404 (2)
N1—C2	1.358 (2)	C13—C14	1.390 (2)
N1—C11	1.4713 (19)	C13—C18	1.508 (2)
C2—C7	1.390 (2)	C14—C15	1.384 (2)
C2—C3	1.411 (2)	C14—H14	0.9500
C3—C4	1.362 (2)	C15—C16	1.393 (2)
C3—H3	0.9500	C15—C19	1.506 (2)
C4—C5	1.419 (3)	C16—C17	1.388 (2)
C4—H4	0.9500	C16—H16	0.9500
C5—C6	1.365 (2)	C17—C20	1.509 (2)
C5—H5	0.9500	C18—H18A	0.9800
C6—C7	1.392 (2)	C18—H18B	0.9800
C6—H6	0.9500	C18—H18C	0.9800
C7—N8	1.387 (2)	C19—H19A	0.9800
N8—O10	1.2842 (17)	C19—H19B	0.9800
N8—N9	1.3166 (18)	C19—H19C	0.9800
C11—C12	1.506 (2)	C20—H20A	0.9800
C11—H11A	0.9900	C20—H20B	0.9800
C11—H11B	0.9900	C20—H20C	0.9800
C12—C17	1.400 (2)		
			
N9—N1—C2	111.49 (12)	C14—C13—C12	118.82 (14)
N9—N1—C11	120.06 (12)	C14—C13—C18	119.21 (14)
C2—N1—C11	128.19 (13)	C12—C13—C18	121.96 (15)
N1—C2—C7	106.08 (13)	C15—C14—C13	122.03 (14)
N1—C2—C3	133.66 (15)	C15—C14—H14	119.0
C7—C2—C3	120.26 (14)	C13—C14—H14	119.0
C4—C3—C2	116.27 (16)	C14—C15—C16	118.30 (14)
C4—C3—H3	121.9	C14—C15—C19	120.77 (15)
C2—C3—H3	121.9	C16—C15—C19	120.92 (15)
C3—C4—C5	122.64 (16)	C17—C16—C15	121.43 (14)
C3—C4—H4	118.7	C17—C16—H16	119.3
C5—C4—H4	118.7	C15—C16—H16	119.3
C6—C5—C4	121.56 (15)	C16—C17—C12	119.41 (14)
C6—C5—H5	119.2	C16—C17—C20	118.91 (14)
C4—C5—H5	119.2	C12—C17—C20	121.67 (14)
C5—C6—C7	115.80 (16)	C13—C18—H18A	109.5
C5—C6—H6	122.1	C13—C18—H18B	109.5
C7—C6—H6	122.1	H18A—C18—H18B	109.5
N8—C7—C2	105.00 (13)	C13—C18—H18C	109.5
N8—C7—C6	131.53 (15)	H18A—C18—H18C	109.5
C2—C7—C6	123.47 (15)	H18B—C18—H18C	109.5
O10—N8—N9	122.37 (13)	C15—C19—H19A	109.5
O10—N8—C7	125.79 (13)	C15—C19—H19B	109.5
N9—N8—C7	111.77 (13)	H19A—C19—H19B	109.5
N8—N9—N1	105.66 (12)	C15—C19—H19C	109.5
N1—C11—C12	113.08 (13)	H19A—C19—H19C	109.5
N1—C11—H11A	109.0	H19B—C19—H19C	109.5
C12—C11—H11A	109.0	C17—C20—H20A	109.5
N1—C11—H11B	109.0	C17—C20—H20B	109.5
C12—C11—H11B	109.0	H20A—C20—H20B	109.5
H11A—C11—H11B	107.8	C17—C20—H20C	109.5
C17—C12—C13	119.98 (14)	H20A—C20—H20C	109.5
C17—C12—C11	120.02 (14)	H20B—C20—H20C	109.5
C13—C12—C11	120.00 (14)		
			
N9—N1—C2—C7	0.47 (17)	C11—N1—N9—N8	−174.86 (13)
C11—N1—C2—C7	174.51 (14)	N9—N1—C11—C12	−35.7 (2)
N9—N1—C2—C3	−179.86 (17)	C2—N1—C11—C12	150.74 (15)
C11—N1—C2—C3	−5.8 (3)	N1—C11—C12—C17	95.32 (17)
N1—C2—C3—C4	−178.70 (17)	N1—C11—C12—C13	−85.41 (18)
C7—C2—C3—C4	0.9 (2)	C17—C12—C13—C14	1.8 (2)
C2—C3—C4—C5	−0.2 (3)	C11—C12—C13—C14	−177.43 (13)
C3—C4—C5—C6	−0.6 (3)	C17—C12—C13—C18	−177.49 (14)
C4—C5—C6—C7	0.6 (2)	C11—C12—C13—C18	3.2 (2)
N1—C2—C7—N8	−0.46 (16)	C12—C13—C14—C15	−1.6 (2)
C3—C2—C7—N8	179.81 (14)	C18—C13—C14—C15	177.75 (14)
N1—C2—C7—C6	178.74 (14)	C13—C14—C15—C16	0.1 (2)
C3—C2—C7—C6	−1.0 (2)	C13—C14—C15—C19	−179.23 (15)
C5—C6—C7—N8	179.14 (15)	C14—C15—C16—C17	1.1 (2)
C5—C6—C7—C2	0.2 (2)	C19—C15—C16—C17	−179.53 (16)
C2—C7—N8—O10	177.40 (14)	C15—C16—C17—C12	−0.8 (2)
C6—C7—N8—O10	−1.7 (3)	C15—C16—C17—C20	−179.64 (15)
C2—C7—N8—N9	0.32 (17)	C13—C12—C17—C16	−0.7 (2)
C6—C7—N8—N9	−178.79 (15)	C11—C12—C17—C16	178.61 (14)
O10—N8—N9—N1	−177.24 (13)	C13—C12—C17—C20	178.11 (15)
C7—N8—N9—N1	−0.04 (17)	C11—C12—C17—C20	−2.6 (2)
C2—N1—N9—N8	−0.28 (17)		

### Hydrogen-bond geometry (Å, °)

**Table:**

Cg3 is the centroid of the C12–C17 ring.

**Table:**

*D*—H···*A*	*D*—H	H···*A*	*D*···*A*	*D*—H···*A*
C6—H6···O10^i^	0.95	2.35	3.190 (2)	147
C16—H16···O10^ii^	0.95	2.58	3.506 (2)	165
C18—H18A···Cg3^iii^	0.98	2.98	3.810 (18)	144

Symmetry codes: (i) −*x*+1, −*y*+1, −*z*; (ii) −*x*+1, *y*−1/2, −*z*+1/2; (iii) −*x*+2, *y*−1/2, −*z*+1/2.
